# Patient socioeconomic determinants of the choice of generic versus brand name drugs in the context of a reference price system: evidence from Belgian prescription data

**DOI:** 10.1007/s10198-012-0377-8

**Published:** 2012-03-18

**Authors:** Maria-Isabel Farfan-Portet, Carine Van de Voorde, France Vrijens, Robert Vander Stichele

**Affiliations:** 1 Belgian Health Care Knowledge Centre (KCE), Boulevard du Jardin Botanique, 55, 1000 Brussels, Belgium; 2 Heymans Institute of Pharmacology, Medical School, University of Ghent, De Pintelaan 185, 9000 Ghent, Belgium

**Keywords:** Pharmaceuticals, Reimbursement, Reference pricing, Socioeconomic factors, Cost-sharing, Belgium, H51, I11, I18

## Abstract

The generic reference price system (RPS) can impose a financial penalty for patients using a brand name drug instead of its generic alternative. Previous studies on the impact of the RPS have not considered the potentially differential effect of using generic alternatives for individuals with a different socioeconomic background. However, patients’ characteristics might determine their overall knowledge of the existence of the system and thus of the financial burden to which they may be confronted. The association between patients’ characteristics and the use of generic drugs versus brand name drugs was analyzed for ten highly prescribed pharmaceutical molecules included in the Belgian generic reference price system. Prescriptions were obtained from a 10% sample of all general practitioners in 2008 (corresponding to 120,670 adult patients and 368,101 prescriptions). For each pharmaceutical molecule, logistic regression models were performed, with independent variables for patient socioeconomic background at the individual level (work status, having a guaranteed income and being entitled to increased reimbursement of co-payments) and at the level of the neighborhood (education). The percentage of generic prescriptions ranged from 24.7 to 76.4%, and the mean reference supplement in 2008 ranged from €4.3 to €37.8. For seven molecules, higher use of a generic alternative was associated with either having a guaranteed income, with receiving increased reimbursement of co-payments or with living in areas with the lowest levels of education. Globally, results provided evidence that the generic RPS in Belgium does not lead to a higher financial burden on individuals from a low socioeconomic background.

## Introduction

Among the various measures employed by European countries to control public spending on pharmaceutical products, reference pricing is one of the most popular. A reference price system (RPS) establishes a common reimbursement or reference price for a group of comparable or interchangeable drugs, called a cluster [1–7]. The third-party payer reimburses no more than the reference price for all drugs within the same group. Therefore, a patient taking a drug whose price is above the reference price has to pay the difference. This extra patient cost is usually referred to as the “reference supplement.” Such a system controls drug expenditures for the third-party payer by (1) making consumers and physicians decrease their demand for relatively higher priced drugs, thus stimulating them to choose less expensive alternatives and (2) stimulating price competition in drug markets [2].

The most controversial issue in the RPS is the definition of the cluster, which can be narrowly or broadly defined. The broader the definition of a cluster, the higher the number of drugs for which the same reference price applies. A system with clusters including only original drugs that lost their patent protection and a possibly long series of generic alternatives for that off-patent original product is called “a generic RPS” or “generic referencing” [8]. This is the system in place in Belgium, Denmark, France, Portugal and Spain [9]. Broader definitions of clusters can be either (1) products with chemically related active ingredients that belong to the same pharmacological class (for instance the class of all drugs containing a statin as active ingredient) or (2) products that may be neither chemically identical nor pharmacologically equivalent, but have comparable therapeutic effects (for instance different types of antihypertensive drugs). Such a system is commonly addressed in the literature as “therapeutic reference pricing” [2]. Typically, countries that have put such a broader version of the RPS in place make use of a mixture of narrow and broad clusters, depending on the types of drugs. The Netherlands, Germany, Italy and Hungary have such a system.

Most studies assessing the impact of the RPS cover its effect on drug use, and changes in price and expenditures [2, 5, 10, 11]. Those studies reported that the introduction of reference pricing was followed by an increase in the use of drugs priced at or below the reference level. Accordingly, the use of the more expensive drugs decreased. A price reduction for both the brand and generic drugs was also observed. The reduction varied among the different studies, but was usually higher for brand drugs than for generic alternatives [5, 8]. Finally, most studies concluded that the introduction of a RPS reduced drug expenditures for the third-party payer. For Belgium, studies on the impact of the RPS are in line with the international literature [12–18]. Indeed, population-based studies reported that the market share of generics increased from 11% in 2001 to around 40% in 2007 and that savings for the third-party payer amounted to €57 million in 2003 [14, 15]. However, it was estimated that in 2008, €60 million in reference supplements was paid by patients on top of the obligatory coinsurance [9].

While there is no doubt that the introduction of the RPS led to financial benefits for patients and the third-party payer, little is known about which patients bear the financial burden related to the reference supplement. Indeed, only one recent study considers the impact of the introduction of a generic RPS on consumer welfare [19]. The authors found that introducing the generic RPS reduced patients’ co-payments via a decrease in prices for both brand and generic drugs. However, the authors relied on aggregated data and thus could not measure the financial burden for patients paying the reference supplement when using a brand name drug. Moreover, only a few studies have considered whether adherence to the RPS depends on patient characteristics [20–22]. Nevertheless, those characteristics might determine whether patients are fully aware of the existence and the consequences of a RPS and consequently have an impact on the choice of drugs. Characteristics such as age, gender and education have previously been considered as important determinants of health behavior [23, 24] as well as of how individuals obtain health information [25]. Thus, if patients are not equally aware of the existence of a RPS, those from the weakest groups in society might end up using more pharmaceuticals for which a reference supplement is due. Therefore, a policy encouraging patient responsibility should be closely monitored to avoid undesirable outcomes in terms of disparities in access to health care according to socioeconomic status.

The aim of this study was to establish whether the generic price reference system, as introduced in 2001 in Belgium, had a detrimental effect on the equity of health care. We explored whether patients from lower socioeconomic classes were prescribed more or less often costly drugs with a supplement penalty within a cluster where cheaper reimbursed medicines existed (generic alternative). Given that pharmacist generic substitution (dispensing a generic drug even if the prescription is for a branded original product) is not allowed in Belgium, the prescription received by the patient determines the drug that will be dispensed by pharmacists. Hence, if prescribed, it is the patient’s responsibility to ask the doctor for a generic alternative and to avoid the reference supplement. Since the RPS is designed in such a way that the financial incentive and the initiative are shifted from the provider to the demand side, such measures expose patients to the financial consequences of their drug use. However, the possible implications of the system on the financial accessibility of different socioeconomic groups have never been evaluated.

This article is structured as follows. In the first part the Belgian RPS is described. Data and methods used in the empirical analysis are provided in the second part. In the third part results are presented. The last section is the conclusion.

## The reference price system in Belgium

Belgium has a compulsory health insurance system with broad coverage including drugs. Pharmaceutical reimbursement decisions are made by the Minister of Social Affairs who is advised by the Drug Reimbursement Committee (CRM/CTG).^1^ The Drug Reimbursement Committee submits advice for a new drug based on the assessment and appraisal of a reimbursement request file sent by a pharmaceutical company. The file includes an overview of the clinical evidence for drugs for which the company claims an added therapeutic value plus an economic evaluation and a proposal for the reimbursement conditions. At the same time, the company asks approval for the requested drug price at the Ministry of Economic Affairs. The CRM/CTG advises the Minister of Social Affairs on the reimbursement basis, which might differ from the requested price. Prices are not capped, but only drugs in class 1 (drugs for which the company claims added therapeutic value) can obtain a price premium. Prices for drugs in class 2 (with similar therapeutic value compared to other drugs) must correspond to prices abroad or to similar products. Finally, generics and copies included in class 3 must set prices applying a substantial rebate on the original price of the brand drug [26].

Reimbursement for new drugs is set as a coinsurance with a ceiling for patients. Coinsurance rates and ceilings differ between patients with and without preferential reimbursement and according to the reimbursement categories (which reflect the therapeutic necessity of the drug). Table 1 presents the cost-sharing schemes in 2008.

**Table 1:** Co-payments for ambulatory drugs in 2008

Reimbursement category	Preferential reimbursement^a^	Non-preferential reimbursement
Category A—Vital drugs (e.g., insulin for diabetics, cancer drugs, antiretrovirals)	No co-payment	No co-payment
Category B—Therapeutically significant drugs for non life-threatening diseases (e.g., antibiotics, antiasthmatics, antihypertensives)	15% with a maximum of €7.20	25% with a maximum of €10.80
Category B—Large package size	15% with a maximum of €8.90	25% with a maximum of €13.50
Category C—Therapeutically less significant drugs for systematic treatment (e.g. antiemetics, spasmolytics)	50% with a maximum of €8.90	50% with a maximum of €13.50
Category Cs—Drugs used for certain chronic illnesses (e.g., drugs for coronary heart disease, antihisthamines, and vaccines)	60% without a maximum	60% without maximum
Category Cx—Contraceptives and antispasmodics	80% without a maximum	80% without maximum

^a^Preferential reimbursement of co-payment is granted to individuals who fall below a certain income threshold. On 1 April 2010 a new cost-sharing scheme for pharmaceuticals was established in order to improve pharmacist’s remuneration while keeping the cost-sharing level of patients constant

*Source*: Vrijens et al. [9]

Reimbursement of pharmaceutical expenses increased in nominal terms by an average of 7.5% per year between 1990 and 2000 compared to an annual rate of 5.1% for total spending on health care [27]. To control pharmaceutical costs a RPS, called nationally the “reference reimbursement system,” was introduced on 1 June 2001 for off-patent reimbursable drugs provided that a generic alternative exists. Initially clusters included only original brands and generic alternatives with the same dosage and the same administration form. During subsequent years inclusion criteria were relaxed in order to enlarge the scope of the RPS. The most important change was made on 1 July 2005 when the definition of the cluster was extended to include all drugs having the same active ingredient (so belonging to the same ATC-5 classification group) [28] independently of dosage and administration routes [9]. Along with the definition of the size of a cluster, the reference price is the second most important component that defines a RPS. In general, countries tend to define the reference price based on the price of all or some drugs included in the cluster [9]. In Belgium, the reference price is based on a simple linear reduction (percentage) in the original ex-factory price of the brand drug. The result is then increased by the distribution and delivery margins to obtain the public price, as is the case for all drugs.

When the RPS was first introduced in 2001, the percentage reduction in the original ex-factory price of the brand drug was fixed at 16% [9, 29]. It was then progressively increased through the years and is currently equal to 30% for drugs included in the RPS for the first time, to 32.80% for drugs included in a reference group for over 2 years and 35.15% for drugs included in a reference group for over 4 years. The reference prices are revised four times a year.

When public drug prices are decreased by national compulsory measures, the reference price is affected and decreased proportionally. For instance, in April 2010, a new cost-containment measure introduced a biannual application of a compulsory price reduction for ‘old’ drugs: drugs reimbursed for over 12 years and less than 15 years had their ex-factory price and reimbursement basis reduced by 15%, and drugs reimbursed for over 15 years underwent a 17% reduction. These reductions imply that the reference price is reduced accordingly, twice a year (1 January and 1 July). Table 2 summarizes the percentage reduction applied to the original brand drug price to obtain the reference price of the cluster. This percentage varies with two factors: the number of years the original drug is included in the RPS and the number of years the original drug is already reimbursed by a third-party payer (TPP).

**Table 2:** Difference between the reference price and the price of the original brand drug

Number of years original drug reimbursed by the TPP^b^	Number of years original drug included in the RPS^a^		
	New in the RPS (%)	In the RPS for >2 years (%)	In the RPS for >4 years (%)
Less than 12 years	30.00	32.80	35.15
12–15 years	40.50	42.88	44.88
>15 years	41.90	44.20	46.20

^a^Reference price system

^b^Third-party payer

*Source*: Vrijens et al. [9]

A consequence of the modalities of the RPS in Belgium is that several pharmaceutical companies manufacturing commonly used original brand drugs reduced their prices to the level of the reference price. As this decreases the price differential between the brand name and the generics, the reference supplement for the patient can be avoided and their market share remains unaffected [30]. Thus, for these clusters in the RPS containing original brand drugs and generic alternatives, there is no financial penalty (the reference supplement) for the patient whatever the choice of drugs. For a total of 10.7 million inhabitants, patients paid in 2008, on top of the obligatory coinsurance, €60 million in reference supplements, corresponding to 10% of total out-of-pocket expenses for reimbursed pharmaceuticals.

Like in many other countries that have implemented a RPS, policymakers recognized the important role of physicians’, pharmacists’ and patients’ behavior in the prescription and use of drugs not incurring the reference supplement. A set of measures for these three actors was introduced to increase the impact of the RPS. Measures for physicians include information campaigns as well as the establishment of minimum percentages of “low-cost” drug prescriptions. The National Drug Information Centre was asked to introduce a color-code schema in its information products (book, web site, computer applications) to indicate very clearly the costly drugs requiring a supplement. Easily accessible price comparisons for all drug groups were published on the website of the Information Center, and the third-party payer (the National Institute for Health and Disability Insurance -RIZIV/INAMI) printed booklets with these price comparisons for all drug groups for free distribution to all physicians. Low-cost drugs include both generic drugs as well as original brand drugs that aligned their prices to the reference price, as described above. To ensure that prescription quotas of low-cost drugs were respected, monitoring of the physician’s prescribing pattern and sanctions for physicians who do not comply with the quotas were also set, although these were rarely implemented [31]. In 2001, the year of the introduction of the RPS, the share of low-cost drugs represented 6.6% of all reimbursed defined daily dose (DDD): 4.2% for generic drugs and copies, and 2.4% for original products that lowered their price to the reimbursement basis. In 2008, the share of low-cost drugs was 40.3% of the DDD (24.0% generics, 16.3% low-cost brand name drugs). Only 11.8% of the total DDD of reimbursed drugs entailed a supplement for the patient [9].

The role of the community pharmacist in the delivery of reimbursed drugs in Belgium is limited to providing information to patients. Contrary to other countries, when faced with a branded prescription, Belgian pharmacists are not legally allowed to substitute an original brand drug for a generic alternative. Only when the prescription is written using the International Nonproprietary Name (INN) of the drug are pharmacists allowed to deliver a low-cost drug. However, prescribing by INN is not widespread in Belgium: only 7% of prescriptions were written in the INN in 2009 [32]. Until recently, the pharmacists received a percentage of the pharmacy retail price (31%, VAT not included). The legislator (Minister of Social Affairs) ensured that pharmacists’ margins were the same in absolute value for both generic and brand drugs. But by triggering price competition, the RPS has indirectly contributed to the erosion of pharmacists’ remuneration. Since 1 April 2010, a new remuneration system for the pharmacist exists. The pharmacist’s remuneration consists of: (1) a fixed payment per delivery (75% of total income); (2) a variable payment as a percentage of the pharmacy ex-factory price (20%); (3) a complementary fixed payment (5%). The fixed payment equals €3.87 and aims to remunerate the drug delivery. The variable payment or economic margin pays for the operating cost of the pharmacy. The complementary fixed payment aims to remunerate specific tasks including deliveries with the INN prescription (€1.20 per delivery). By limiting the share of the economic margin in the total remuneration, the new system partially disconnects the pharmacist’s profit margin from the retail price.

Besides the financial penalty (i.e., the reference supplement) that patients pay when choosing a brand name drug instead of a generic alternative, public authorities and sickness funds launched some rather limited information campaigns to encourage adherence to the RPS.

## Methods and data

### Study design and sampling

To study the link between patients’ socioeconomic characteristics and the use of generic drugs versus brand name drugs incurring a reference supplement in the context of the Belgian generic RPS, a cross-sectional design and a two-step sampling procedure were used. The first step of the sampling procedure consisted of selecting a random sample of 10% of all prescribing general practitioners (GPs) in Belgium. To exclude occasional prescribers, prescribers with fewer than 200 prescriptions in 2008 were not included in the sample. The second step was to select all adult patients having received at least one prescription from one of the physicians selected in the first step. Only adult patients were included because the drugs selected in the analysis are indicated for adults (and are given exceptionally to children for very specific indications). For all selected individuals (patients and physicians) detailed information was obtained, including demographic and socioeconomic characteristics, and information on all the pharmaceutical products prescribed in an ambulatory setting. For each pharmaceutical product received by a patient in the sample, the co-payment as well the reimbursement by the third-party payer was obtained. Information not available at an individual level (income and education) was obtained for the smallest geographical unit (statistical sector) of the patient’s residence.

The selection of drugs was based on two criteria. First, only clusters for which the choice between a brand and a generic drug incurred a reference supplement for the patient were included. Second, the restriction to commonly prescribed clusters^2^ had to guarantee a sufficient sample size. Molecules in our selection of clusters do not have a narrow therapeutic margin, and thus switching patients from the brand drug to the generic alternative does not pose a health risk for the patient [33].

### Data

#### Databases

The study period covered all pharmaceuticals reimbursed during 2008. Data were extracted from several administrative databases. First, individual patient data were obtained from the Intermutualistic Agency (IMA), a non-profit institution that collects information from all sickness funds in Belgium [29]. Variables used in the analysis are from three databases (collected by the IMA) that are linked by the encrypted beneficiary number. Patients’ demographic and socioeconomic characteristics are from the Population data set and information on pharmaceutical products delivered in community pharmacies (not in the hospital) from the Pharmanet database. Pharmanet contains exhaustive information on pharmaceutical dispensing, including the reimbursement category, number of packages, insurance reimbursements, co-payments, date of dispensing and prescriber identification number. Other reimbursed acts (not relating to pharmaceutical products) are registered in the Health Care database. Information on patients having a medical record with their GP, patients residing in a rest or nursing home for the elderly, and patients enrolled in a primary care center financed by the lump sum was selected from this data set. Because these data were not available for the year 2008, we relied on data for 2007. Finally, because data on income and education were not available at the level of the individual patient, we used data at the level of the statistical sector (SS). Statistical sectors divide municipalities into homogeneous entities according to several criteria making them reflect similar “neighborhoods” in terms of socioeconomic, urban and morphological characteristics. Statistical sectors vary in size; sometimes they are not larger than a street or a neighborhood. Data on education at the level of the statistical sector are based on the 2001 Census [34], and data on income are available from the tax administration for the year 2005. For the sample of prescribers in 2008, the third-party payer provided information on gender and age.

#### Demographic and socioeconomic characteristics

##### Patients’ characteristics

Age and gender for all patients were obtained from the IMA data set. For the purpose of the analysis, four age groups were created (18–44; 45–64; 65–74; 75 and more). Six additional dummy variables describe other patients’ characteristics: living in a residential long-term institution for the elderly, receiving a guaranteed income, being entitled to increased reimbursement of co-payments, receiving a lump sum for chronic illness, being inscribed in a medical care center and having a global medical record with the GP. A more detailed description of these variables is provided in the Appendix. Entitlement to a guaranteed income and to increased reimbursement of co-payments is conditional on a low income. Thus, these variables serve as a proxy for low socioeconomic background. The variables “being inscribed in a medical care center” and “having a global medical record” are included to capture patient loyalty to his/her physician. Patients can opt for having a global medical record held by a particular GP. In return they receive increased reimbursement for their primary care. Patients can also choose to enroll in a primary care center financed per capita and in return have free access to primary care. Patients’ work status was categorized into four categories: unemployed, employee, self-employed worker, and invalid or handicapped. Finally, being entitled to a lump sum for chronic illness is an indirect indicator of health status.

##### Physician’s characteristics

As generic substitution is not allowed in Belgium, physicians’ characteristics were included as control variables. For age, a categorical variable regrouping individuals in four age groups was created (25–34; 35–44; 45–54; 55 and older). Gender was represented by a dummy variable (1 for male).

##### Geographic information

Five income groups were created based on median taxable income for 2005. The education level of the statistical sector of the patient was aggregated using the International Standard Classification of Education (ISCED) [35]. We used the share of individuals having attained post-secondary education (ISCED 4 and 5) over the total population aged 18 years and older (see Table 3).

**Table 3:** Lower and upper limits to define income and education quintiles of each statistical sector (SS-small area information)

Quintile	Income (2005) limits in € (based on SS median income)		Adults who attained post-secondary education (2001) (%)	
	Lower limit	Upper limit	Minimum	Maximum
Q1	682	16,450	0	13.78
Q2	16,451	18,611	13.79	18.80
Q3	18,612	20,310	18.81	23.57
Q4	20,312	22,305	23.58	30.10
Q5	22,306	57,195	30.11	100

In addition, a patient’s region of residence was included in the model to control for unobservable regional characteristics. Region of residence corresponds to Flanders (57.8% of population), Wallonia (32.4%) or Brussels (9.8%).

#### Statistical analysis

The observation unit was the prescription. As Belgium opted for a generic reference price system, use of brand drugs and their generic alternatives must be considered within each cluster. Indeed, as physicians do not have to choose among different active ingredients (as in a therapeutic reference price system), we consider that for each specific molecule the decision is limited to whether or not to use a generic alternative. The dependent variable in each model was a dummy variable with a value equal to 1 if the patient was prescribed a generic drug and 0 otherwise (i.e., using a brand drug and paying the reference supplement). Logistic regression models were used to assess associations between patients’ characteristics and the probability of being prescribed a generic drug. The method of Generalized Estimating Equations (GEE) [36] was applied to adjust the variance of each parameter estimate for the clustering of prescriptions within patients. As this method limits the clustering to one level, variance estimates for physicians’ characteristics, as well as those of small area characteristics might be underestimated. All factors (except the income variable) described in the previous section were included in the final model, whether statistically significant or not. This choice was made to allow proper comparisons of effects across all drugs analyzed. Odds ratios and 95% CI were derived from these regression models. *P* values presented are those of the effect of the factor as a whole (i.e., testing if there is any difference between all levels of the factor) and not *P* values from pairwise comparisons (testing each level of the factor to a reference category).

Analysis of the model robustness revealed collinearity problems between the two small area characteristics, income and education. In our sample, correlation between these two factors equaled 0.6. Sensitivity analyses revealed that the education level was more discriminatory than the income level, and thus only the education level of each patient’s small area was used in the final models (tables including income are available from the authors).

## Results

### Selection of patients, prescribers and pharmaceutical products

The random sample of 10% of all prescribers corresponded to 826 GPs and to a total of 402,407 patients. For these patients, 1,526,084 prescriptions corresponded to clusters where a choice between a brand name drug with a reference supplement and generic alternatives existed. A total of 66 different clusters distributed in 7 anatomical main groups (ATC-1) were identified (tables for all molecules are available from the authors). The analysis was further restricted to commonly prescribed clusters covering a wide range of anatomical main groups and indications. Our final database contained a total of 368,101 prescriptions and 120,670 patients distributed in ten different clusters.

### Descriptive results for the ten selected molecules

The ten molecules selected for the analysis were lansoprazole, glicazide, furosemide, bisoprolol and thiazides, diltiazem, clarithromycin, piroxicam, tramadol, citalopram and acetylcysteine. The lowest percentage of generic prescriptions is for piroxicam (20.9%) and the highest is for citalopram (76.4%). The mean annual reference supplement also varied considerably among the different molecules from €4.3 for acetylcysteine to €37.8 for diltiazem (see Table 4).

**Table 4:** Generic prescription and the reference supplement for the ten molecules included in the study

ATC-1 Level	Analysis groups (molecules and ATC-5 group)	Generic prescription			Reference Supplement				
		Total	Generic	%	Patients paying		Amount (in €)		
					N	%	Mean	Median	Maximum
A—Alimentary tract and metabolism	1. Proton pomp inhibitor								
	Lansoprazole	12,057	7,782	*64.5*	884	22.8	32.9	27.1	91.8
	2. Anti-diabetic								
	Gliclazide	16,849	11,890	*70.6*	978	35.6	19.4	15.4	96.5
C— Cardiovascular system	3. Diuretic								
	Furosemide	42,827	16,212	*37.9*	9,564	64.3	6.1	3.3	209.9
	4. Beta blockers								
	Bisoprolol and thiazides	57,849	35,964	*62.2*	3,650	27.1	18.2	16.4	65.8
	5. Antihypertensives								
	Diltiazem	34,125	8,425	*24.7*	3,911	67.7	37.8	36.3	161.6
J—Antiinfectives for systemic use	6. Quinolone antibacterials								
	Clarithromycin	19,438	13,797	*71*	4,581	28.8	8.7	7.6	180.3
M—Musculo-skeletal system	7. Anti-inflammatory drugs								
	Piroxicam	36,393	7,596	*20.9*	18,614	81.0	8.3	5.9	156.9
N—Nervous system	8. Analgesic								
	Tramadol	67,332	24,100	*35.8*	13,904	67.4	16.6	7.3	726.0
	9. Antidepressant								
	Citalopram	25,567	19,535	*76.4*	1,365	17.7	31.8	21.7	187.7
R—Respiratory system	10. Mucolytic								
	Acetylcysteine	55,664	25,351	*45.5*	19,955	54.5	4.3	3.2	146.4

**Fig. 1:**
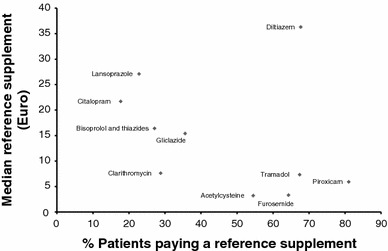
Percentage of patients paying a reference supplement and median reference supplement (in €) for ten molecules

Figure 1 includes the median reference supplement incurred by patients in 2008 and the percentage of patients who actually paid it for the ten molecules in our sample. The expected relation between both variables is in theory simple: a high reference supplement should dissuade patients from buying a brand drug. Indeed, in a generic reference price system, drugs are considered interchangeable in terms of benefits and risks for the patients; thus, only the price of each drug should play a role in determining which drug to use. However, patient preference as well as physician’s prescription habits may also determine the extent to which generic alternatives are used instead of the more expensive brand [37, 38].

We found that for lansoprazole and citalopram a small percentage of patients using the brand name drug end up paying a high reference supplement. Indeed, differences in the price between brand and generic alternatives (for certain administration forms) reach €9.21 for lansoprazole and €13.04 for citalopram per package. However, for diltiazem, the molecule having the highest median reference supplement in our sample, this result was not found. A high percentage of patients used the brand drug for diltiazem and thus ended up paying the reference supplement. Our results also show that the percentage of patients using the brand drug was high for some pharmaceuticals for which the median reference supplement paid in 2008 was small (piroxicam, furosemide and acetylcysteine). For these molecules, a difference in the price between brand and generic alternatives per package was €6.2, €2.47 and €3.22 for piroxicam, furosemide and acetylcysteine, respectively. A possible explanation might be that the price difference between the brand drug and the generic alternatives is too small to guide consumption behavior. However, patients might also be more sensitive to differences in prices (even if they are small) for pharmaceuticals used for chronic conditions.

### Regression results

Table 5 presents the results of the analysis for the ten molecules. To simplify the interpretation of our results, we try to identify trends in the association between patient characteristics and the probability of using a generic alternative for the ten clusters.

**Table 5:** Association between the use of generic alternatives and patient and physician characteristics

Variable	Variable	Category	A. lansoprazole		A. gliclazide		C. furosemide		C. bisoprolol and thiazides		C. diltiazem	
			OR	*P* value	OR	*P* value	OR	*P* value	OR	*P* value	OR	*P* value
Patients’ characteristics	Gender (ref female)	Male	0.95 (0.91–0.99)	0.012	1.01 (0.97–1.05)	0.636	1.01 (0.99–1.04)	0.197	1.00 (0.97–1.02)	0.705	1.02 (1.00–1.05)	0.079
	Age group (ref 18–44)	45–64	0.94 (0.88–1.01)	0.397	0.96 (0.80–1.16)	0.816	1.00 (0.94–1.06)	0.447	1.08 (1.03–1.13)	0.002	0.99 (0.90–1.08)	0.003
		65–74	0.95 (0.86–1.04)		0.98 (0.81–1.19)		0.98 (0.91–1.06)		1.09 (1.03–1.16)		1.01 (0.91–1.11)	
		75+	0.94 (0.85–1.04)		0.96 (0.79–1.16)		0.97 (0.90–1.04)		1.12 (1.06–1.18)		0.95 (0.86–1.05)	
	Patient receives lump sum for chronic illness	Yes	1.01 (0.94–1.09)	0.768	0.95 (0.88–1.02)	0.145	0.98 (0.96–1.00)	0.117	1.09 (1.04–1.15)	≤0.001	0.94 (0.91–0.97)	≤0.001
	Patient has guaranteed income	Yes	1.04 (0.93–1.17)	0.5	1.02 (0.93–1.13)	0.667	1.00 (0.95–1.05)	0.939	1.07 (1.00–1.14)	0.043	0.94 (0.89–0.99)	0.033
	Entitled to increased reimbursement	Yes	1.00 (0.94–1.06)	0.98	1.00 (0.95–1.05)	0.924	1.04 (1.02–1.06)	≤0.001	1.02 (0.99–1.05)	0.134	1.00 (0.97–1.03)	0.824
	Work status (ref pensioners)	Invalids and handicapped	1.00 (0.90–1.10)	0.279	1.08 (0.99–1.18)	0.138	1.02 (0.97–1.08)	0.002	0.96 (0.91–1.02)	0.043	1.02 (0.96–1.08)	0.131
		Unemployed	1.07 (0.98–1.17)		0.94 (0.85–1.04)		1.02 (0.97–1.08)		0.99 (0.95–1.04)		1.07 (1.01–1.14)	
		Employee	1.08 (1.00–1.17)		0.96 (0.87–1.08)		1.10 (1.03–1.17)		0.95 (0.91–0.98)		1.06 (1.00–1.12)	
		Self–employed worker	1.04 (0.91–1.19)		0.92 (0.75–1.13)		0.92 (0.84–1.00)		0.98 (0.92–1.04)		1.02 (0.94–1.10)	
	Patient in a primary care center	Yes	1.10 (0.95–1.27)	0.231	1.03 (0.91–1.17)	0.602	1.31 (1.21–1.42)	≤0.001	1.22 (1.13–1.33)	≤0.001	0.97 (0.90–1.05)	0.469
	Patient has a global medical record	Yes	0.96 (0.91–1.00)	0.057	1.15 (1.09–1.21)	≤0.001	1.05 (1.02–1.07)	≤0.001	1.03 (1.01–1.06)	0.018	1.07 (1.05–1.10)	≤0.001
	Patient in rest or nursing home	Yes	1.23 (1.06–1.43)	0.008	0.93 (0.82–1.07)	0.328	1.10 (1.07–1.13)	≤0.001	1.00 (0.90–1.10)	0.927	0.98 (0.93–1.04)	0.502
Physicians’ characteristics	Physician gender (ref female)	Male	1.07 (1.01–1.14)	0.025	0.97 (0.91–1.03)	0.284	1.02 (1.00–1.05)	0.096	1.05 (1.01–1.08)	0.004	1.01 (0.98–1.04)	0.596
	Physician age group (ref ≤35)	36–45	0.99 (0.90–1.09)	0.073	0.95 (0.86–1.05)	0.515	0.95 (0.91–0.99)	≤0.001	0.92 (0.88–0.96)	0.006	1.08 (1.03–1.14)	≤0.001
		46–55	1.00 (0.91–1.10)		0.98 (0.89–1.07)		0.97 (0.93–1.01)		0.94 (0.90–0.98)		1.04 (1.00–1.09)	
		55+	0.93 (0.85–1.03)		0.99 (0.91–1.09)		0.91 (0.87–0.95)		0.95 (0.91–0.99)		0.97 (0.92–1.01)	
Geographic Information	Education (ref Quintile 1)	Q2 education	1.02 (0.96–1.09)	0.952	0.99 (0.93–1.05)	0.525	0.97 (0.94–1.01)	≤0.001	0.97 (0.94–1.01)	0.049	0.99 (0.96–1.03)	0.77
(Patient residence)		Q3 education	1.02 (0.95–1.08)		1.01 (0.95–1.07)		0.97 (0.94–1.00)		1.00 (0.97–1.04)		0.98 (0.95–1.02)	
		Q4 education	1.00 (0.93–1.07)		0.96 (0.90–1.03)		0.95 (0.92–0.98)		0.98 (0.95–1.02)		1.00 (0.96–1.04)	
		Q5 education	1.01 (0.94–1.09)		0.95 (0.88–1.04)		0.92 (0.89–0.95)		0.95 (0.92–0.99)		0.98 (0.94–1.02)	
	Geographic region (ref Brussels)	Flanders Wallonia	0.89 (0.80–0.98) 0.90 (0.82–0.99)	0.042	1.27 (1.17–1.37) 1.08 (0.99–1.19)	≤0.001	1.18 (1.14–1.23) 1.18 (1.14–1.23)	≤0.001	1.04 (0.98–1.10) 0.93 (0.88–0.99)	≤0.001	1.15 (1.11–1.20) 1.12 (1.08–1.17)	≤0.001

Variable	Variable	Category	J. clarithromycin		M. piroxicam		N. tramadol		N. citalopram		R. acetylcysteine	
			OR	*P* value	OR	*P* value	OR	*P* value	OR	*P* value	OR	*P* value
Patients’ characteristics	Gender (ref female)	Male	1.02 (1.01–1.04)	0.007	1.03 (1.01–1.05)	≤0.001	1.01 (0.98–1.03)	0.509	1.03 (0.99–1.06)	0.099	1.02 (1.01–1.04)	≤0.001
	Age group (ref 18–44)	45–64	0.99 (0.97–1.01)	0.451	0.97 (0.95–0.99)	≤0.001	0.96 (0.92–1.00)	≤0.001	0.97 (0.93–1.02)	0.446	1.01 (0.99–1.03)	≤0.001
		65–74	0.97 (0.93–1.01)		0.93 (0.90–0.96)		0.93 (0.88–0.99)		0.95 (0.89–1.02)		0.97 (0.94–1.00)	
		75+	0.97 (0.92–1.01)		0.94 (0.91–0.98)		0.89 (0.84–0.95)		0.95 (0.89–1.01)		0.95 (0.92–0.99)	
	Patient receives lump sum for chronic illness	Yes	1.03 (0.98–1.08)	0.225	1.00 (0.96–1.03)	0.902	0.97 (0.94–1.00)	0.049	0.98 (0.94–1.02)	0.367	0.96 (0.94–0.98)	≤0.001
	Patient has guaranteed income	Yes	1.05 (0.99–1.10)	0.098	1.00 (0.95–1.06)	0.873	1.03 (0.97–1.10)	0.277	1.07 (1.00–1.15)	0.043	1.02 (0.98–1.07)	0.326
	Entitled to increased reimbursement	Yes	1.00 (0.97–1.03)	0.896	1.04 (1.01–1.06)	0.002	1.01 (0.98–1.03)	0.714	1.04 (1.00–1.07)	0.043	1.02 (1.01–1.04)	0.011
	Work status (ref pensioners)	Invalids and handicapped	0.99 (0.95–1.03)	0.091	1.01 (0.97–1.05)	0.223	0.97 (0.93–1.02)	0.07	1.01 (0.95–1.07)	0.464	0.97 (0.93–1.00)	0.01
		Unemployed	1.01 (0.97–1.04)		1.02 (0.99–1.06)		1.04 (0.98–1.09)		1.01 (0.95–1.07)		1.02 (0.99–1.05)	
		Employee	1.03 (1.00–1.06)		1.00 (0.97–1.03)		0.97 (0.92–1.02)		1.02 (0.97–1.08)		0.99 (0.96–1.01)	
		Self-employed worker	1.00 (0.96–1.05)		1.03 (0.99–1.07)		1.02 (0.92–1.13)		0.93 (0.84–1.02)		1.00 (0.96–1.04)	
	Patient in a primary care center	Yes	0.92 (0.85–1.00)	0.061	1.31 (1.19–1.44)	≤0.001	1.15 (1.08–1.23)	≤0.001	1.09 (1.02–1.17)	0.015	1.17 (1.11–1.24)	≤0.001
	Patient has a global medical record	Yes	1.02 (1.00–1.04)	0.013	1.07 (1.05–1.09)	≤0.001	1.04 (1.01–1.07)	0.002	1.06 (1.03–1.10)	≤0.001	1.02 (1.01–1.04)	0.004
	Patient in rest or nursing home	Yes	1.09 (1.02–1.16)	0.009	0.99 (0.90–1.08)	0.749	0.98 (0.94–1.03)	0.44	1.07 (1.02–1.12)	0.007	0.91 (0.87–0.94)	≤0.001
Physicians’ characteristic	Physician gender (ref female)	Male	0.99 (0.97–1.01)	0.171	1.06 (1.04–1.09)	≤0.001	1.01 (0.98–1.04)	0.394	0.95 (0.92–0.98)	0.004	0.93 (0.92–0.95)	≤0.001
	Physician age group (ref ≤35)	36–45	1.05 (1.02–1.09)	≤0.001	1.01 (0.97–1.04)	≤0.001	0.95 (0.90–0.99)	0.005	0.97 (0.92–1.03)	≤0.001	0.99 (0.96–1.01)	≤0.001
		46–55	1.02 (0.99–1.06)		0.97 (0.94–1.01)		0.99 (0.94–1.03)		1.04 (0.99–1.09)		0.97 (0.95–1.00)	
		55+	0.99 (0.96–1.02)		0.93 (0.90–0.96)		0.95 (0.91–1.00)		0.97 (0.92–1.02)		0.95 (0.92–0.97)	
Geographic Information	Education (ref Quintile 1)	Q2 education	0.98 (0.96–1.01)	≤0.001	0.99 (0.97–1.01)	≤0.001	0.99 (0.95–1.02)	0.056	0.98 (0.94–1.02)	0.034	1.01 (0.99–1.03)	≤0.001
(Patient residence)		Q3 education	0.97 (0.95–1.00)		1.00 (0.98–1.03)		0.99 (0.96–1.03)		0.96 (0.92–1.00)		1.04 (1.02–1.06)	
		Q4 education	0.95 (0.92–0.98)		0.97 (0.94–0.99)		0.96 (0.93–0.99)		0.96 (0.92–1.00)		1.04 (1.02–1.06)	
		Q5 education	0.95 (0.92–0.97)		0.93 (0.91–0.96)		0.95 (0.92–0.99)		0.93 (0.88–0.97)		1.05 (1.02–1.08)	
	Geographical region (ref Brussels)	Flanders Wallonia	1.04 (1.01–1.08) 1.14 (1.11–1.18)	≤0.001	1.11 (1.08–1.15) 1.00 (0.97–1.03)	≤0.001	1.07 (1.03–1.11) 1.03 (0.99–1.07)	0.002	1.09 (1.04–1.15) 1.08 (1.03–1.14)	0.005	1.02 (0.99–1.05) 1.01 (0.98–1.04)	0.548

#### Patient and physician demographic characteristics

Patient and physician demographic characteristics were introduced as control variables. The role of patient age and gender in the use of a brand versus a generic drug within the context of the reference price system is not straightforward. The association between patient age and gender and the probability of using a generic drug was found to be rather heterogeneous over the ten molecules. While for five active ingredients (diltiazem, clarithromycin, piroxicam, citalopram and acetylcysteine) men had a higher probability than women of receiving a generic drug, for lansoprazole the opposite result held. For the other molecules, gender did not play a role in using a generic drug. These results are in line with previous studies where patient characteristics such as gender, age and ethnicity have little or no association with the use of generic drugs [38, 39]. The age of the patient even had a less pronounced association with the probability of using a generic drug. For three drug clusters (piroxicam, tramadol and diltiazem) patients aged 18–44 years were more likely to use a generic alternative. For the combination of bisoprolol and thiazides, younger patients had a smaller probability of using a generic alternative.

The same conclusion holds for the association between physician age and gender and the probability of using a generic drug. Physician gender played a role in prescribing behavior in six groups. Male doctors prescribed more generics for lansoprazole, furosemide, bisoprolol and thiazides, and piroxicam, and less for citalopram and acetylcysteine. Although physician age was also associated with prescribing behavior, no clear pattern was identified for the ten molecules.

#### Patient health status

Although the separate analysis for the ten molecules increased the homogeneity of patient health status within each group, differences remain. No direct health status information was available in the databases. However, entitlement to a lump sum for chronic illness can be interpreted as an indirect measure of health status. The variable was included as a control variable. Compared to more healthy individuals, patients receiving a lump sum for being chronically ill were less likely to use a generic alternative for diltiazem, tramadol and acetylcysteine. The opposite held for bisoprolol and thiazides.

#### Patient socioeconomic characteristics

Patient socioeconomic background was proxied by three variables available at the individual level and one at the level of the statistical sector. For the individual characteristics—work status, having a guaranteed income and being entitled to increased reimbursement—some patterns can be observed. Having a guaranteed income was associated with a higher probability of using a generic version for bisoprolol and thiazides, clarithromycin and citalopram, and a lower probability for diltiazem. This last result is rather striking, since diltiazem has the highest reference supplement (see Fig. 1, Table 4). Higher use of a generic alternative of furosemide, piroxicam, acetylcysteine and citalopram was also associated with patients being entitled to increased reimbursement.

Work status was associated with the use of a generic drug for five active ingredients (furosemide, bisoprolol and thiazides, claritromycin, tramadol and acetylcysteine) but with opposite results. Compared to pensioners, which is our reference category, the probability of using a generic version of furosemide was higher for the other work status categories. On the contrary, for bisoprolol and thiazides, pensioners were more likely to use its generic version than all other groups. For clarithromicyn, tramadol and acetylcysteine, invalids and handicapped patients were less likely to use a generic alternative than pensioners.

Patients living in small areas with low education levels were more likely to use a generic alternative for six types of drugs: furosemide, bisoprolol and thiazides, clarithromycin, piroxicam, tramadol and citalopram. Only for acetylcysteine the opposite result was found: individuals living in more educated areas were more likely to use generic alternatives.

#### Patient choice within the health system

The two variables reflecting patient loyalty to his/her physician were associated with a higher probability of using generic alternatives in several groups of drugs. Patients enrolled in a primary care center were more likely to receive generic alternatives in six groups: furosemide, bisoprolol and thiazides, piroxicam, tramadol, citalopram and acetylcysteine. Patients having a global medical record were more likely to be prescribed generic versions in nine out of the ten groups (except for lansoprazole). A possible explanation for the higher generic drug use in primary care centers could be that generic prescribing is an essential part of their policy [40]. Moreover, there is some evidence that the type of practice may influence prescription behavior of physicians [39]. A possible explanation for the larger probability of generic drug use for patients with a global medical record might be that, since having a medical record is possible only if patients ask their preferred GP to keep one, these patients have a better knowledge of the health system, including the existence of the reference supplement and how to avoid it. Another hypothesis is that patients and physicians using such a tool might be better suited to discuss prescription choices and thus use the less expensive alternatives.

#### Rest and nursing home for the elderly

Residing in a rest or nursing home for the elderly was associated with a higher use of generic alternatives for lansoprazole, furosemide, clarythromicin and citalopram. For acetylcysteine, the opposite result was found.

#### Regional characteristics

Compared with individuals living in Brussels, those living in Flanders and Wallonia were more likely to use generic alternatives in seven out of the ten groups that were analyzed (glicazide, furosemide, diltiazem, clarithromycin, piroxicam, tramadol and citalopram).

## Discussion

There are many different approaches to evaluate a reference price system. A number of studies have tried to evaluate its effect on outcome measures such as drug use, changes in prices and cost for the third-party payer and for patients. Only a few studies have directly assessed its impact on financial accessibility [20–22]. However, a reference price system might impose a larger financial burden on more disadvantaged individuals if their knowledge of the existence and consequences of the system is not the same as that of more privileged individuals [41].

To our knowledge, this is the first article directly analyzing the possible unintended differential impact of a generic reference price system on individuals with a different socioeconomic background. Several results are worth mentioning. First, the most stable results for the association of patient characteristics and the probability of using a generic drug were found for variables reflecting patient loyalty to his/her GP. However, both variables could also be interpreted as reflecting a patient’s sensitivity to the cost of care. Patients with a medical record benefit from a 30% reduction in the co-payment of a GP consultation. Patients enrolled in a primary care center do not pay any co-payments. Maybe these patients are more informed on how to reduce their health care expenditures. In addition to this, one peculiarity of the pharmaceutical market is that the demand for pharmaceutical specialties is not determined solely by patients, but instead jointly by patients, prescribers and pharmacists. Having a medical record as well as being enrolled in a primary care center might also reflect how patients and physicians interact. Indeed, if those tools allow doctors to better internalize the health and financial cost for patients, they might result in a more efficient prescription behavior.

We did not identify a consistent pattern between the use of a generic drug within clusters in the RPS and patients’ socioeconomic characteristics. There are a number of significant differences for individual drugs, and specific subgroups, but their clinical relevance can be questioned. Furthermore, the characteristics of the drugs may also be important: drugs for acute (hence short) use, drugs for serious complex conditions (e.g., diltiazem in cardiac patients, mostly prescribed initially by the cardiologist) and drugs perceived as strong brands (gliclazide, furosemide, piroxicam, acetycysteine). However, except for diltiazem, either a positive relationship, or none at all, between the use of a generic drug and a lower socioeconomic status was found. In terms of financial accessibility, the generic price reference system in Belgium seems to work well.

Although the results do not provide evidence that the RPS imposes an unbalanced financial burden on low-income patients, some patients are still bearing the cost of using brand drugs when a cheaper alternative is available. How can it be explained that there are still prescriptions entailing a reference supplement? This question is particularly important in a system of generic reference pricing with narrowly defined clusters where potential differences in clinical effectiveness of generic and brand drugs can be regarded as negligible. Some hypotheses can be put forward. First, no nationwide information campaign directed towards the general public was organized. Second, generic substitution by pharmacists is not allowed. In Belgium, the pharmacist can dispense a low-cost medication only when prescriptions are written using the International Non-proprietary Name. Currently, only 7% of all prescriptions use the INN. Third, for physicians the time investment necessary to have a sound knowledge of the rapid changes in prices as well as their prescription habits may create a barrier for prescribing the less expensive alternatives [37–39]. Finally, the perception of generic drugs by GPs may play an important role. A number of surveys have been carried out in Belgium on this topic. Vrijens et al. [9] provide an overview of these surveys and conclude that the perception of generic drugs among GPs is positive, but that the reasons why they do not prescribe them are varied: price reasons (when the original has the same price as the generic alternative), because specifically asked for by patients, certain reticence about quality, or because it concerns very specific therapeutic indications. Furthermore, patients show a high degree of confidence in their physician. Even though they are aware of the existence of generic drugs and overestimate the price differential, they hesitate to ask their prescriber to change the prescription. The extent to which a reference price system is able to attain both the policy objective of controlling expenditures as well as being equitable essentially relies on the interaction among the physician, the patient and the pharmacist. Measures in Belgium have mostly been directed toward patients and physicians. A step further may be to allow pharmacist generic substitution. In addition to this, to avoid inequities among patients, introducing a cost-sharing measure that intends to provide patients with monetary incentives to alter their consumption behavior should be accompanied by measures increasing patients’ awareness of the financial consequences of this behavior.

## Appendix

See Table 6.

**Table 6:** Definition of the variables describing patient characteristics

Receiving a guaranteed income	This variable is equal to one for individuals receiving the minimum guaranteed income or receiving assistance from a public municipal welfare center
Entitled to increased reimbursement of co-payments	An increased reimbursement for physicians’ visits, hospitalizations as well as for pharmaceuticals is granted to individuals who fall below a certain income threshold
Receiving a lump sum for chronic illness	The lump sum compensates for extra expenses accompanied by a chronic illness. Two conditions have to be fulfilled at the same time. First, the amount of co-payments exceeds a threshold during 2 consecutive years. A second condition concerns the degree of dependency during the current calendar year
Enrollment in a primary care center	Patients can choose to enroll in a primary care center (“maison medicale in French, wijkgezondheidscentrum in Dutch”). Contrary to the majority of GPs who are paid fee-for-service, primary care centers are financed by lump sum. Patients do not pay any co-payments
Having a global medical record	Patients can choose to open a global medical record. In this case, their GP creates a record containing data on their health such as chronic illness, current treatments, etc. Having a global medical record reduces fees for GP’s visits

## Footnotes

1 Le Polain et al. [26] provide a detailed description of the decision process that takes place for the reimbursement of a new drug.

2 Within our large database, we selected the most prescribed pharmaceutical molecules. More precisely, lansoprazole and gliclazide were the most prescribed for the alimentary tract and metabolism group. For the cardiovascular system, we selected the most prescribed diuretic (furosemide), beta-blocker (bisoprolol and thiazides) and antihypertensive (diltiazem). Among quinolones, clarithromicine was the most prescribed. Piroxicam was the most prescribed antinflammatory drug that is not delivered over the counter. Citalopram, tramadol and acetylcysteine were the most prescribed antidepressant, analgesic and mucolytic.
